# Nanotechnology Based Approaches in Phage Therapy: Overcoming the Pharmacological Barriers

**DOI:** 10.3389/fphar.2021.699054

**Published:** 2021-10-05

**Authors:** Sandeep Kaur, Anila Kumari, Anjana Kumari Negi, Vikas Galav, Shikha Thakur, Manish Agrawal, Vandana Sharma

**Affiliations:** ^1^ Department of Food Science, Mehr Chand Mahajan DAV College for Women, Chandigarh, India; ^2^ Department of Biochemistry, Dr. Rajendra Prasad Government Medical College, Himachal Pradesh, India; ^3^ Department of Veterinary Pathology, Post Graduate Institute of Veterinary Education and Research (RAJUVAS), Jaipur, India; ^4^ Department of Biotechnology, Kumaun University, Uttarakhand, India

**Keywords:** nanotechnology, phage therapy, nano-emulsification, encapsulation, liposome, nanofibers, pharmacological barriers

## Abstract

With the emergence and spread of global antibiotic resistance and the need for searching safer alternatives, there has been resurgence in exploring the use of bacteriophages in the treatment of bacterial infections referred as phage therapy. Although modern phage therapy has come a long way as demonstrated by numerous efficacy studies but the fact remains that till date, phage therapy has not received regulatory approval for human use (except for compassionate use).Thus, to hit the clinical market, the roadblocks need to be seriously addressed and gaps mended with modern solution based technologies. Nanotechnology represents one such ideal and powerful tool for overcoming the pharmacological barriers (low stability, poor in-vivo retention, targeted delivery, neutralisation by immune system etc.) of administered phage preparations.In literature, there are many review articles on nanotechnology and bacteriophages but these are primarily focussed on highlighting the use of lytic and temperate phages in different fields of nano-medicine such as nanoprobes, nanosensors, cancer diagnostics, cancer cell targeting, drug delivery through phage receptors, phage display etc. Reviews specifically focused on the use of nanotechnology driven techniques strictly to improve phage therapy are however limited. Moreover, these review if present have primarily focussed on discussing encapsulation as a primary method for improving the stability and retention of phage(s) in the body.With new advances made in the field of nanotechnology, approaches extend from mere encapsulation to recently adopted newer strategies. The present review gives a detailed insight into the more recent strategies which include 1) use of lipid based nano-carriers (liposomes, transfersomes etc.) 2) adopting microfluidic based approach, surface modification methods to further enhance the efficiency and stability of phage loaded liposomes 3) Nano- emulsification approach with integration of microfluidics for producing multiple emulsions (suitable for phage cocktails) with unique control over size, shape and drop morphology 4) Phage loaded nanofibers produced by electro-spinning and advanced core shell nanofibers for immediate, biphasic and delayed release systems and 5) Smart release drug delivery platforms that allow superior control over dosing and phage release as and when required. All these new advances are aimed at creating a suitable housing system for therapeutic bacteriophage preparations while targeting the multiple issues of phage therapy i.e., improving phage stability and titers, improving *in-vivo* retention times, acting as suitable delivery systems for sustained release at target site of infection, improved penetration into biofilms and protection from immune cell attack. The present review thus aims at giving a complete insight into the recent advances (2010 onwards) related to various nanotechnology based approaches to address the issues pertaining to phage therapy. This is essential for improving the overall therapeutic index and success of phage therapy for future clinical approval.

## Introduction

Phages are ubiquitous in nature representing the most abundant organisms in our ecosystem playing an important part in regulation and development of microbial communities (Clokie et al., 2011; Sharma et al., 2017). Like their host bacteria, phages have been exploited by the research community in many different fields ranging from phage display, cancer cell targeting, drug delivery, diagnostic applications, gene delivery, and nanoprobes etc. (Harada et al., 2018). One of the important properties of phages that had been explored long back but has showed a renewed interest is “Phage Therapy” i.e., the application of lytic phages against pathogenic bacteria leading to their lysis and final killing (Sulakvelidze et al., 2001; Lin et al., 2017). This application of phages also called phagotherapy represents a potential antibacterial strategy worth exploring and the reasons are many fold. The primary and the strongest reason is the need to develop methods to fight the battle against rising antibiotic resistance. Overuse and misuse of antibiotics globally has led to the emergence and spread of Antimicrobial resistance, 2020 (AMR) as we are stepping close to the threat of entering the post-antibiotic era (Zucca and Savoia, 2010; Ventola et al., 2015; Taneja et al., 2019). WHO has declared AMR in the list of top 10 global public health threats facing humanity (Antimicrobial resistance, 2020).This calls for the scientific community to focus all its efforts to develop novel and promising non-antibiotic options. Phage therapy is definably a potent non-antibiotic option to curtail the relentless increase in antibiotic-resistant bacteria and all efforts in making this treatment enter the clinical market are prudent (Gordillo Altamirano and Barr, 2019; Brives and Pourraz, 2020). Secondly, phages are omnipresent in nature and they are natural commensals of human and animal body (Kutter and Sulakvelidze 2005; Clokie et al., 2011; Koskella and Brockhurst, 2014; Batinovic et al., 2019). Their ubiquitous nature strongly supports the fact that they are harmless entities showing no ill effects to eukaryotic cells even at high titers. Next major advantage is their high specificity and a targeted killing mechanism. Lytic phages infect their target bacterial cells after recognizing specific receptors. This specificity allows attacking target bacterial strains only further making this option a safe and gentle approach (Loc-Carillo et al., 2011; Principi et al., 2019). Besides these points, phages exhibit the property of self-dosing as they are able to replicate at the expense of host bacterium. This contributes to regulate phage numbers in direct relation with pathogen level allowing their diffusion in nearby areas till the pathogen population is present (Abedon and Thomas-Abedon, 2010; Drulis-Kawa et al., 2012).

Phage therapy has shown significant potential as highlighted by various *in vitro* and *in vivo* studies done in the past against range of bacterial infections. Results indicate that this therapy has immense potential with applications in human medicine as well as veterinary science, agriculture, and food sector. Despite this picture, the fact remains that till date, and phage therapy has not received regulatory approval for human use (except for compassionate/emergency use and in the food sector). Although phage therapy has come a long way, but there are still major loopholes and gaps that need to be addressed and worked upon with modern solution based technologies (Alvarez et al., 2019; Nilsson, 2019).

Nanotechnology is one such ideal and powerful tool for overcoming majority of the barriers associated with administered phage preparations. The present review firstly provides an overview of the major pharmacological and clinical challenges acting as major hurdles. The review then focuses on discussing in detail, the different nanotechnology based advances (extending from mere nano-encapsulation to the more recent strategies adopted) along with their mode of action in improving the pharmacological aspects and benefits thus observed. The discussion is supported by documenting recent *in-vitro* and *in-vivo* research studies done and the gaps addressed. The present review thus tends to give a comprehensive understanding into the integration and exploitation of nanotechnology based modelling in phage therapy for overcoming and optimising the pharmacokinetic profile of phage and phage products.

## Pharmacological Barriers in Phage Therapy: an Overview

Pharmacology of any administered drug can be divided into two domains: 1) pharmacokinetics and pharmacodynamics. Briefly, pharmacodynamics includes the therapeutic effects of the administered drug on the body while pharmacokinetic profile of the drug dictates its absorption, retention and clearance within the patient’s body (Baggot, 1990; Palleria et al., 2013). Both these domains are inter-dependent and help to define an ideal drug and its therapeutic index and this stands true for phages as well. Looking at the pharmacokinetic aspect of phage administration, the unmet limitations are many and even more than antibiotics. Firstly, we need to admit the fact that phages are living entities (unlike chemical drugs) showing large variations among themselves depending on their virulence, differences in lytic infection cycle such as burst size, adsorption rate, and latent period etc. (Abedon et al., 2001; Gallet et al., 2011; Nilsson, 2019). Moreover, phages are bigger molecules in terms of size than a normal chemical drug which makes its uptake and translocation within the body more challenging and more susceptible to be easily cleared by the innate system. This makes phage pharmacokinetics fundamentally different from the normal chemical drugs (Drulis-Kawa et al., 2012; Navarro and muniesa, 2017). Second criterion which decides the therapeutic efficacy is the requirement of high titers of phage (i.e., maintenance of viability) at the target site so as to dominate over the bacterial population and control their multiplication. This is the most difficult achievable parameter as it depends on number of factors which are further linked to each other. These include: 1) route of administration, 2) target bacterial density 3) *in-vivo* clearance rates (clearance by immune cells, phagocytes, complement, and reticuloendothelial system), 4) inactivation of active phages (low pH, bile, body fluids, and enzymes etc.) and 5) poor penetration and inaccessibility of phage to reach deep seated tissue areas and biofilm based infections while crossing all barriers (Payne and Jansen, 2001,^2003^; Levin and Bull, 2004; Dąbrowska and Abedon, 2019).

It is well known that body’ impact to any drug varies with the mode of drug administration (Benet, 1978; Jin et al., 2015). Phages have been administered via many routes such as topical application, intraperitoneal (*i.p*), subcutaneous (*s.c*), intramuscular (*i.m*), intravenous (*i.v*) as well as oral delivery. For example, giving phage via injections helps to overcome body’s innate defence barriers allowing the phage to rapidly reach systemic circulation showing higher therapeutic effect especially while treating immediate conditions like bacteremia or septicaemia etc. (Bogovazova et al., 1991; Chhibber et al., 2008; Jennes et al., 2017). Same way, the *i. p* route is more effective in delivering high titers of phage reaching blood circulation sooner than when given via *i. m* or *s. c* routes (McVay et al., 2007; Takemura-Uchiyama et al., 2014). Also, directly injecting the phage to the target tissue or direct application onto the wound site seems the most favourable option as systemic circulation is further bypassed and distribution obstacles to phage movement are minimised (Beridze, 1938; Kumari et al., 2009; Chhibber et al., 2013; Yin et al., 2017; Ferry et al., 2018) However, there exist challenges regarding delivery of phages *via* oral route for treating enteric infections or *via* inhalation route in case of treating respiratory infections. Oral administration of phage is faced by major barriers as phage struggles to bear the harsh environment of the gastrointestinal tract. High stomach acidity, bile intolerance and inactivation by digestive enzymes and other proteases lead to significant drop in final titre not sufficient enough to produce the intended therapeutic effect (Young and Gill, 2015; Sarker et al., 2016; Abdelsattar et al., 2019). Similarly, as compared to *i. p*. and *i. m*. injections in animal models, inhalation has resulted in poor absorption efficacy (Carmody et al., 2010; Singla et al., 2015).

For phage therapy to be effective, the dose of phage present at the infection site must be higher than the inundation threshold (i.e., concentration of free phage required for reducing bacterial loads). For this, phage titers need to be maintained at high numbers through productive infection that is further dependant on target bacterial density as a minimal bacterial concentration (called proliferation threshold) is needed for this (Payne et al., 2000; Payne and Jansen, 2001; Abedon, 2009; Ryan et al., 2011; Abedon 2014). Further, to maximise this effect, phages need to be applied as close to site of infection where active bacterial population is high enough to bring exponential increase in phage numbers *via* repeated lytic cycles (Nilsson, 2014). But this is not the case with most of the routes. Thus, it becomes a major challenge to protect and maintain phage titers (as they pass across various barriers of human body) till they reach the target site.

Another challenge is the inaccessibility of phage to reach deep seated infection areas as such sites are complexed due to poor penetration and presence of dead tissue debris, necrotic cells, immune cells, tissue gluing material etc. (Dąbrowska and Abedon, 2019; Huh et al., 2019). Further, phages being small lipophilic molecules do not exhibit efficient transdermal absorption and penetration for use in deep seated skin infections (Dąbrowska, 2019).

Although phages display potent anti-biofilm ability, there does exists penetration and motion barriers through the biofilm matrix. As the biofilm matrix matures and becomes thick, its interior become less favourable to virion diffusion and maintaining active phage infection. Also, while moving, phages meet adsorbed biofilm bacteria (not the free-living ones) and such adsorbed bacteria fail to support productive phage infections (Gonzalez et al., 2018; Simmons et al., 2018). Another obstacle is the inaccessibility of phage to infect the intracellular pathogens as phage lack the ability to enter eukaryotic cells. Studies in past have shown that phages when co-infected with their target bacterium showed reduction in intracellular loads but phage alone was unable to bring the same effect (Capparrelli et al., 2007; Kaur et al., 2014) thus emphasizing on the need of an optimal phage delivery vehicle to treat such intracellular infections.

Finally, the biggest issue is phage inactivation and short half-life due to clearance from the patient’s body leading to low clinical benefit. Since phages are large molecules, they are easily trapped and cleared by the mononuclear phagocyte system (MPS). Both liver and spleen are active organs of the MPS system and are responsible for phage neutralisation and early exit from the body (Abedon and Thomas-Abedon 2010; Dabrowska and Abedon, 2019). This explains that after intravenous administration, the titers rapidly drop to significantly low levels, mainly due to innate immunity and MPS clearance as noted by past researchers in different animal models (Schultz and Frohlich., 1965; Inchley, 1969; Oechslin et al., 2017). Other nonspecific factors include complement mediated clearance, inactivation by body fluid’s components and enzymes etc. leading to phage inactivation and the same was observed in various studies leading to rapid loss of viable titers (Sulkin et al., 1957; Keller and Zatzman, 1959; Majewska et al., 2015; Bichet et al., 2021).

Finally, phage neutralisation is not just due to nonspecific responses but also due to specific induction of antibody response which is subject to route of application and dosing regimen. Phage administration through local application evokes with minimal immune reaction but phages administered *via* systemic routes have a tendency to evoke a higher immune reaction. The presence of antibodies induced by administration of phages has been reported in animal studies (Uhr et al., 1962; Huff et al., 2002; Huff et al., 2010; Majewska et al., 2015) as well as in humans studies (Slopek et al., 1987) and these neutralising antibodies can be devastating for phage therapy especially when the treatment regimen needs recurring phage applications (Fogelman et al., 2000). With these challenges, some of the human trials did not progress to the next stage and were halted owing to failures and loopholes at one or the other front as depicted in Table 1.

**TABLE 1:** Major human trials for testing efficacy of phage therapy and reason of failures.

Year	Brief details	Major limitations/findings	References
June 2009 to September 2011	Prospective, single center, randomized, placebo-controlled trial to study the safety and efficacy of T4-like phage cocktail in 6–24-month-old male children presenting acute diarrhoea.	• Oral coliphages showed a safe gut transit in children	Sarker et al. (2016)
		• Failed to achieve intestinal amplification attributed to too low *E. coli* pathogen titers not enough to support active phage infection.	
		• Possible contribution of other pathogens such as *Streptococcus spp*. as causative agents	
2014	• Study on 122 patients at the Phage Therapy Unit in Wrocław with bacterial infections to verify whether phage therapy (PT) can induce anti-phage antibodies when give through various routes (orally, locally, orally/locally, intrarectally, or orally/intrarectally).	• Phage inactivation rates (K value) showed low K values (≤1.73) seen in sera of healthy volunteers and also low K value in patients before PT (K ≤ 1.64). However, in 12.3% of examined patients (*n* = 15), phages given locally (*n* = 13) and phages given locally/orally (*n* = 2) showed high K values in their sera i.e., K > 18 as measured 15–60 days of PT and high phage inactivation was seen especially in patients treated with some *S. aureus*, *P. aeruginosa*, and *E. faecalis* phages	Łusiak-Szelachowska et al. (2014)
	• Anti-phage activity determined in sera from the 122 patients before and during PT, and in sera from 30 healthy volunteers using a neutralization test. Further, levels of anti-phage antibodies determined in sera of 19 patients PT and sera of 20 healthy volunteers using ELISA.		
		• Phage inactivation by sera depends on route of administration and phage type.	
2004	Double-blind, controlled clinical trial to evaluate the efficacy and safety of a therapeutic bacteriophage preparation (Biophage-PA) targeting antibiotic-resistant *P. aeruginosa* in chronic otitis in 24 patients	• Phage therapy showed clearing of infection but only in 3 out of 12 cases while there was only a minor reduction in bacterial counts in other participants indicating towards use of low phage dose (2 × 10^4^ PFU) given as one reason.	Wright et al. (2009)
July 2015 to Jan 2017	Randomised, controlled, double-blind phase trial to study efficacy and tolerability of a cocktail of 12 bacteriophages (PP1131) to treat burn wounds infected by *P. aeruginosa* as compared to standard of care antibiotic	• Trial was halted prematurely due to poor efficacy of phage cocktail mix compared to standard of care (SOC) antibiotic.	Jault et al. (2019)
		• PP1131 titre decreased after manufacturing and participants were given a lower concentration of phages than initially expected.	
		• Ancillary study showed that the bacteria isolated from patients with failed PP1131 treatment were resistant to phage doses.	

Among the major pharmacodynamic limitations, purity of final phage preparation is important as it should be free from all possible impurities and endotoxin. Also, complete characterisation of the therapeutic phage along with its genome annotation is essential so as confirm that the phage in question does not encode for antibiotic resistance or toxins etc. (Philipson et al., 2018; Hyman, 2019). In addition to this, phage host range and the emergence of bacterial resistance to the administered phage are other main factors affecting the therapeutic efficacy and clinical outcome of phage therapy (Dabrowska and Abedon, 2019; Nilsson, 2019). The present review however primarily focuses on the role of nanotechnology based techniques to address most of the pharmacokinetic limitations associated with phage administration. Solutions for overcoming the pharmacodynamic challenges (such as narrow host range, resistance emergence) are presently out of the scope of this review.

## Nanotechnology to the Rescue

The phage–bacterium population densities have a major impact on the successful resolution of bacterial infection by the administered phage (Kasman et al., 2002; Abedon et al., 2011). Although, high phage densities are needed in order to arrest the growth of phage susceptible bacteria, sustained phage amplification is equally essential to further contain the bacterial multiplication and spread. But, phage amplification is further dependent on the concentration of the bacteria and therefore, a minimum threshold density of bacteria at target site is essential for this (Payne and Jansen, 2003; Tsonos et al., 2014). However, if low starting concentrations of bacteria are present or if phage is given at very early stages of infection, it make take some time for the bacteria to grow before reaching the threshold levels to support efficient phage amplification (Cairns et al., 2009; Malik et al., 2017). In such situations, phages may face early decay and drop in initial titer. Also, phages are equally susceptible to be cleared easily by the host immune factors, enzymes, and other tissue fluids or stress factors leading to low viable titter resulting in poor phage therapy outcomes (Dąbrowska, 2019; Principi et al., 2019).

In such situations, encapsulation offers an effective strategy showing potential of serving multiple purposes. The advantages range from protection and shielding of phage from outer environment, acting as an active delivery platform, sustained release of viable phages over a prolonged period etc. (Choińska-Pulit et al., 2015; Abdelsattar et al., 2019; Loh et al., 2020) Thus, encapsulation helps to maintain the phage titers to therapeutically effective levels over a significant time period so that once the bacterial threshold is attained, phage titers can further amplify leading to faster and effective resolution (Malik et al., 2017). Moreover, with modern nanoscale based encapsulation techniques, there has been a significant advancement in addressing many of the issues related to phage therapy. This section discusses the major advancements in encapsulating techniques towards phage therapeutics while supporting each new strategy with proof of data from *in vitro* and *in-vivo* studies reported lately.

### Encapsulation in Lipid Based Nanovesicles

Lipid based nanovesicles represent an attractive and versatile drug delivery approach consisting of vesicle made from natural or synthetic lipids. These nanocarriers self-assemble and self-enclose to form spheres of lipid bilayers with an inner aqueous core ideal for encapsulating and protecting the sensitive drug inside (Puri et al., 2009; Bozzuto and Molinari 2015). Liposomes are one such category which has shown high biocompatibility with phages without affecting their efficiency and numbers during and post the encapsulation process (Singla et al., 2015; Chadha et al., 2017). Liposomes has a number of advantages that favour its use and further exploitation in phage delivery to target site with focus against gut pathogens, intracellular pathogens or biofilm associated pathogens etc. Liposomes aids in shielding phages from the action of the outer stress factors such as low pH of the gastric system during intestinal transit, clearance from reticuloendothelial system (RES), action of neutralising antibodies thus enhancing the *in vivo* circulation time (Takeuchi et al., 2005; Singla et al., 2015; Colom et al., 2015; Singla et al., 2016; Chhibber et al., 2018; Leung et al., 2018). Moreover, studies have shown that liposome encapsulation allowed phage to gain access even to the intracellular pathogens which is a serious drawback with free phages (Nieth et al., 2015). Besides being non-toxic and non-immunogenic, liposome are easy to synthesize and the encapsulation techniques provide full control over the physical parameters such as vesicle size, encapsulating efficiency, and encapsulation dose etc. (Malam et al., 2009; Mallick and Choi, 2014; Lamichhane et al., 2018). Liposomes mimic biological membranes in terms of their structure, behaviour and fluidity and this allow them to undergo various conformational and dynamic transitions which are essential for many biological functions. For example, they can penetrate various host tissue barrier layers such as epidermal layers, intestinal mucosa, and deep seated tissue, even the bacterial mature biofilm areas allowing deeper delivery of the entrapped drug to such sites (Meers et al., 2008; Pierre and Dos, 2011; Hua, 2015; Rukavina and Vanic, 2016).

Liposome formation mostly follows classical techniques including electroformation, thin film hydration, sonication, solvent dispersion, evaporation, hydration or swelling etc., (Wagner and Vorauer-Uhl, 2011; Sawant and Torchilin, 2012). An advancement allowing finer tuning and better control of liposome production is the microfluidics method (van Swaay and deMello, 2013; Carugo et al., 2016). “Microfluidic” refers to the systems that process small quantities of fluids having dimensions at the microscale level in a geometrically constrained volume. This process allows production protocol to be miniaturised on chips and small devices thus giving higher throughput and analytical performance with reduced sample and reagent volumes (Duncombe et al., 2015; Wang et al., 2018; Murphy et al., 2017). Since less reaction volumes are used, the associated cost significantly decreases. Leung et al. (2018) investigated the use of microfluidic flow focusing to encapsulate *Pseudomonas* phages, PEV2 and PEV40. For this, a mixture of soy phosphatidylcholine and cholesterol (4:1) in absolute ethanol was injected into inner channel of a cross mixer followed by injection of the phage suspension from side channels. The resultant liposome sizes so formed varied as per the phage types. The size of liposomes encapsulating PEV2 was in the range of 135–218 nm while liposomes for *Myovirus* PEV40 was bigger i.e., 261–448 nm. The encapsulation efficiency of PEV2 was 59% and that for PEV40 was 50% obtained at a total flow rate of 160 μl/ml and organic/aqueous flow rate ratio (FRR) of 2:3. Therefore, with this technique the encapsulation efficiency was enhanced (higher than that obtained with thin film hydration or extrusion method) while increasing the organic/aqueous flow rate ratio (FRR) with minimal titer reduction for both the phages. FRR has been identified as variable in the microfluidic process, with the highest impact on liposome size, poly-dispersity index (PI) and transfection efficiency (Kastner et al., 2014) and this can be controlled easily using the microfluidics approach. Similarly, Cinquerrui et al. (2018) also investigated the use of a novel co-flow microfluidic glass capillary device for the encapsulation of two model bacteriophages (*E. coli* T3 podovirus and *Staphylococcus* phage K) in sub-micron sized liposomes. The authors were able to develop reproducible yield of sub-micron sized liposomes but the maximum attainable yield of encapsulated of T3 phage was affected by aggregation. Also, most of *S. aureus* phage.

K was found bound to the outside of the formed liposome instead of being trapped inside them. Henceforth, more work is required for further exploitation of this approach. Still, microfluidics represents a robust and high-throughput method for easy scalability and high reproducibility for development of size-controlled liposomes.

Another advancement in liposomal based delivery systems include the surface modification of liposomes i.e., conjugation of suitable polymers on liposomal surface (dextran, chitosan, alginate or synthetic polymer such as polyethylene glycol (PEG), poly (vinyl alcohol) (PVA) etc. This enables to confer properties such as increased circulation time escaping RES inactivation or neutralisation from antibodies etc. (Akbarzadeh et al., 2013; Salmaso and Caliceti, 2013). PEGylation of liposomes has been used by past studies in an attempt to produce nanocarriers called stealth liposomes that are able to evade immune attack by macrophages while reducing the uptake process by the RES (Immordino et al., 2006; Allen and Cullis, 2013; Najlah et al., 2019). The polymer conjugation helps to sterically inhibit the various hydrophobic and electrostatic interactions with plasma proteins or cells. This helps in reducing the liposomal uptake further improving the blood-circulation times essential for treating systemic infections (Harris et al., 2001; Immordino et al., 2006; Lombardo et al., 2016). Moreover, PEG on the surface results in a hydrophilic surface chemistry that allows unobstructed diffusion of such liposomes more easily through the epithelium (Malik et al., 2017). A recent one of its kind study by Otero et al. (2019) studied the bio-distribution of orally administered using flourochrome labelled phages encapsulated within liposomes using non-invasive *in vivo* imaging methodology to monitor their accumulation in stomach and intestinal tract employing athymic nude mouse model. Liposomes so prepared were less than 500 nm in size. To further extend the stability during intestinal transit and circulation, cholesterol and cholesteryl polyethylene glycol 600 Sebacate (Chol-PEG600) were incorporated during the synthesis process. Results showed that liposomes encapsulation of phage resulted in a significant increase of the labelled phages in the mouse stomach, detected even 6 h after oral administration with no significant decrease observed. However, non-encapsulated and encapsulated phages were similar when visualised in intestine. This prolonged persistence of liposomal phages in the stomach advocates their use for targeting enteric infections.

Many intracellular pathogens (*M. tuberculosis, S. enterica*) are able to survive and multiply well within the body’s mononuclear phagocyte system (MPS) system. Bacteriophage encased liposomes is an appealing approach to treat as liposomes can accumulate in MPS due to their interaction with serum proteins (Bitounis et al., 2012; Bozzuto and Molinari, 2015) It has been shown that uptake by MPS for liposomes is enhanced when phosphatidylserine incorporating PEG is used (Malik et al., 2017). Further, Agrawal and Gupta (2000) reported the use of tuftsin, a tetra-peptide, which when conjugated with liposome can enhance the uptake of liposome by MPS and drug loaded tuftsin liposomes. This approach may prove highly effective against intracellular pathogens *(Mycobacterium tuberculosis*). Although phage loaded tuftsin-bearing liposomes has not been tested but this is a promising approach to enable lytic phage or phage cocktail reach the intracellular bacteria and contain the infections process.

Transfersomes are another lipid based nanocarriers that are much more flexible and adaptable. This high flexibility allows them to squeeze even through pores much smaller than their own diameter (Walve et al., 2011; Darwhekar et al., 2012). Due to their low toxicity, higher flexibility, transfersomes represent a better option for transdermal delivery than liposomes for treating deep skin infections (Sachan et al., 2013). Chhibber et al. (2017) reported that transfersome-entrapped phage cocktail showed better persistence and stability than free phages. Also, rats treated with the transfersome-entrapped phage cocktail resolved the experimental MRSA thigh infections within a period of 7 days, unlike the 20-day period required for untreated animals.

Besides whole phages, phage lysins are also a potential approach in the antibacterial campaign representing a step ahead. Endolysins, a class of phage borne proteins are the natural hydrolytic enzymes responsible for the final lysis allowing release of progeny phages from the host bacteria. This they do so by acting on the bacterial cell wall and hydrolysing the peptidoglycan layer (Schmelcher et al., 2012; Love et al., 2020). These cell wall breaching enzymes show direct, instant killing, lacking the issues of associated resistance. Endolysins, being proteins and showing labile enzymatic activity suffer for similar or even more additional barriers than parent phages which include issue of stability, retention of their lytic spectrum at the infection site*, in-vivo* half-life etc. (Gondil and Chhibber, 2021). However, unlike phage encapsulation, endolysin delivery systems based research especially involving use of nanotechnology is still in the early stages. In one recent study, Silva et al. (2021) reported the successful encapsulation of MSlys endolysin in deformable liposomes against *S. pneumoniae*, with appreciable encapsulation efficiencies of ∼35%, high stability and preservation of lytic potency seen during storage at 4°C over a test period of 1 year. Cytotoxicity studies also supported the biocompatible nature of MSlys-loaded liposomes while the endolysin loaded liposomes showed significant anti-pneumococcal activity against both planktonic and biofilm cells.

**FIGURE 1:**
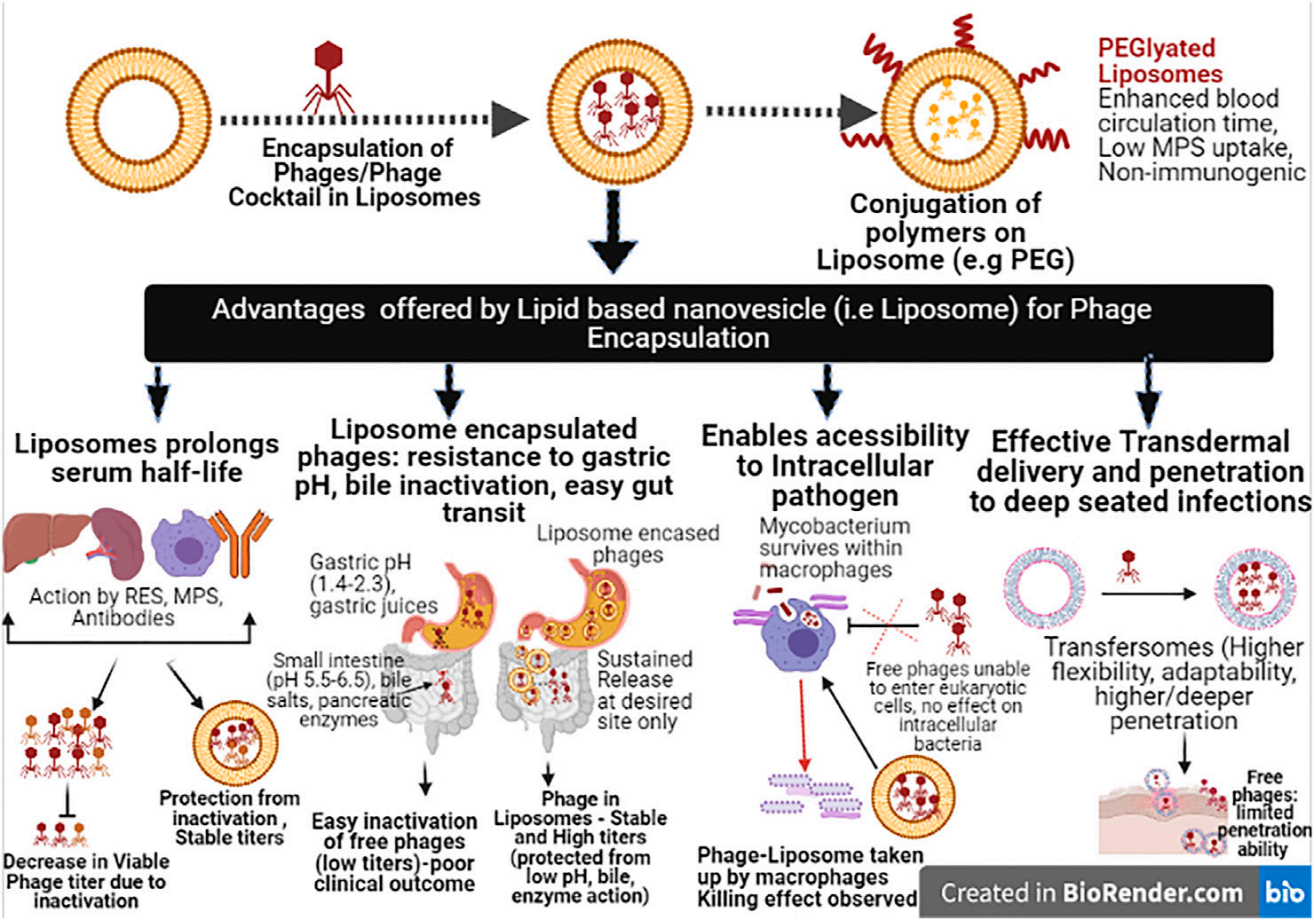
Illustration detailing the advantages offered by Phage Encapsulation in Lipid based nanovesicles (RES, Reticuloendothelial system; MPS, Mononuclear phagocyte system; PEG, Polyethylene glycol).

Similar past studies based on use of liposome and nano-based lipid carriers for encapsulating various phage endolysin showing improved stability and enhanced killing activity was observed unlike non-encapsulated endolysin (Gonzalez Menendez et al., 2018; Bai et al., 2019; Portilla et al., 2020). Table 2 details some of the major studies of both phages and phage encoded lysins benefitted from nano-encapsulation techniques. Figure 1 illustrates and summarizes the advantages offered by use of lipid based nanovesicles while overcoming the associated challenges.

**TABLE 2:** Recent studies focussed on use of Lipid Based Nanocarriers for improved delivery of therapeutic phage and phage endolysins.

Study objective	Study highlights	Desired outcome	References
To evaluate the potential of liposome encapsulation of three phages of different morphologies in reducing gut load of *Salmonella* in poultry.	• Three phages (UAB_Phi20, UAB_Phi78, and UAB_Phi87) active against *Salmonella Typhimurium* were encapsulated in liposomes at a conc. of 10^11^ PFU/ml using the thin film hydration method and this was followed by their lyophilisation to study long term storage stability. Also the phage-liposomal preparation as tested for its titer reduction at simulated gastric fluid (SGF); pH 2.8.	• Phage cocktail encapsulated in the liposomes (raning in size from 308–325 nm) showed appreciable encapsulation efficiency i.e., 49% (UAB_Phi20), 47% (UAB_Phi87), 48% (UAB_Phi78).The titers post-lyophilisation showed high counts in encapsulated phaegs than free phage cocktail i.e., 82% and 84% vs. 22 and 47% for encapsulated and non-encapsulated UAB-Phi20 and UAB-Phi87, respectively, but UAB_Phi78 showed sensitivity.	Colom et al. (2015)
	• Next, the *in vivo* residence time of orally administered liposome phage cocktail (10^10^ PFU/animal) was studied over 72 h and compared with free phage cocktail given to different group.	• *In vivo* residence time results showed prolonged retention of encapsulated phages than free phages in test animals after 48 and 72 h. The percentage (%) of chickens with phage shedding in cecum was 90.5% at 48 h vs. only 38% in free phage treated animals and 38.1 vs. 9.5% at 72 h.	
	• Finally, the *in –vivo* efficacy of phage treatment was studied in the newly hatched broilers by first infecting them (orally on day 0) with 10^7^ CFU/animal of *S*. Typhimurium 14,028 Rifr followed by oral administration of liposome phage cocktail (10^10^ PFU/animal) and free phage cocktail to respective groups given daily for 8 days.	• The encapsulated phages showed prolonged sustained effect in reducing the *Salmonella* gut load even after ceasing the administration. Signficant decline in gut load was seen by day 8 (reduction of 3.8 log CFU) and continued till day 15 (reduction by 1.5 log CFU) as compared to free phage group.	
To evaluate the potential of liposomes encapsulated phages in treating *Klebsiella pneumoniae* induced burn wound infection.	• Phage cocktail of five different phages (KØ1, KØ2, KØ3, KØ4, and KØ5) in an equal proportion (1:1:1:1:1). were encapsulated in cationic liposomal formulation and subjected to complete characterisation.	• Cationic phage loaded liposomes of 230 nm size and low poly-dispersity index (PI of 0.259) and high encapsulation efficiency of 79% were obtained.	Chadha et al. (2017)
	• Experimental acute burn wound infection was established with *K. pneumoniae* (10^5^ CFU/ml) followed by intraperitoneal administration of liposome loaded phage cocktail (LCP-10^5^ PFU/ml i.e MOI-1) to study its therapeutic potential in resolving wound infection in comparison to free phage cocktail (CP; non-encapsulated).	• Control animals showed high bacterial load of ∼8 logs in skin while CP treated showed an initial 4 log count at 72 h further showing a decline. However, minimal counts not exceeding 2 logs was seen with LCP treated mice at 72 h. Also, LCP treated mice showed peak phage titer up to 5 logs within 24 h in the affected skin site.	
		• Also, liposomal phage cocktail was able to protect all test animals even when therapy was delayed by 24 h.	
To study the protective effect of encapsulation of *S.aureus* bacteriophage phiIPLA-RODI in three kinds of nanovesicles	Bacteriophage phiIPLA-RODI encapsulated in three kinds of nanovesicles (niosomes, liposomes, and transfersomes).	• All three types of preparations exhibited high phage encapsulation efficiency (62–98%).	Gonzalez Menendez et al. (2018)
		• Bacteriophage titers and infectivity was stable during 6 months of storage at 4°C with decreases in phage titer below 2 log units for all three types of nanovesicle. The stability of free phages was lower than that of encapsulated phages in all the formulations tested.	
		• Niosome loaded phages were stable even at low pH 4.5 with only a reduction of ∼2 log units from initial titer of 5.76 log PFU/ml.	
To develop and study the efficacy of endolysin encapsulated within cationic liposomes against Gram-negative enteric pathogens.	• Phage-derived endolysin BSP16Lys was isolated, characterized, and then encapsulated into a cationic liposome system.	• Liposome encapsulated endolysin showed significant encapsulation efficiency i.e., 35.27%.	Bai et al. (2019)
	• This was followed by testing its efficacy in bacterial reduction assays.	• Also the encased enzyme showed reduction in *Salmonella Typhimurium* and *E. coli* cell counts by 2.2-log CFU/ml and 1.6-log CFU/ml, respectively, without requiring any membrane permeabilizing agent.	
To evaluate the potential of encapsulating *S. aureus* specific endolysin LysRODI in pH sensitive liposomes	• Endolysin LysRODI was purified and encapsulated (conc. 100 μg/ml) in pH sensitive liposomes that release their content at pH value < 5.5.	• Encapsulated lysin was fully active after its release form the nano-capsules and showed an encapsulation efficacy of 47%.	Portilla et al. (2020)
	• This was followed by testing their encapsulation efficacy and killing activity *via* turbidity assay, time kill curves and anti-biofilm potential against many *S. aureus* strains.	• Antibacterial results showed that in time kill curves, reduction of 2 log units was seen post 60 min of incubation with *S. aureus* Sa9 cells (initial titer of 5.5 log CFU). Also, in co-incubation with exponential Sa9 cells at pH 5.0, greatest level of killing was seen within 30 min (1.85 log units)	
		• The encapsulated endolysin exhibited significant killing activity (2 log units) against biofilm cells of different strains of *S. aureus* at pH 5.0.	
To evaluate therapeutic potential of liposome entrapped phage cocktail (MR-5 and MR-10) to resolve MRSA-induced diabetic excision wound infection.	• Wide spectrum MRSA phages (MR-5 and MR-10) were used as cocktail mix [1:1] and further encapsulated in liposomal vesicles followed by characterisation studies.	• Uniform cationic liposomes with phage cocktail (MR-5 and MR-10) were formed of uniform size (230 nm) an low PI-0.220 and high encapsulation efficiency close to 87%.	Chhibber et al. (2018)
	• Wound excision model with 10^8^ CFU/50 µl of locally injection of *S. aureus* 43,300 (MRSA strain) developed in diabetic mice.	• Liposomes showed high stability over 9 week period at 4°C with no aggregation seen (no change in PI values).	
	• Cocktail loaded liposomal preparations (1:1) and free phage cocktail were then administered locally (10^10^ PFU/ml) at the MRSA infected wound site in diabetic mice followed by studying wound healing parameters and wound bioburden.	• *In vivo* efficacy results showed that in untreated mice, wound bio-burden went up to 8–9 log CFU/ml while in free cocktail group, although there was decline of 3 logs by day 3 but minimal load persisted even upto day 10.However, in liposomal phage cocktail treated mice, highly significant reduction of 4 log CFU was seen by day 3 and negligible counts by day with visible reduction in wound size seen by day 7.	
		• Complete resolution of induced wound infection and healing occurred within 9 days with liposome preparation unlike 20 days for control group.	
To encapsulate mycobacteriophage in liposomes and to study the uptake by eukaryotic cells	• Two model phages i.e., mycobacteriophage TM4 or the reporter bacteriophage λeyfp were encapsulated in giant unilamellar liposomes gel-assisted film hydration method and inverse emulsion.	• Encapsulated phages were taken up more efficiently by both cultured and phorbol 12-myristate 13-acetate (PMA) differentiated THP-1 macrophages than free bacteriophages and able to co-localize with early- and recycling endosomes.	Nieth et al. (2015)
	• Further, the uptake of encapsulated phages was studied by immunofluorescence staining and confocal.	• This showed that such liposomes after their uptake by eukaryotic cells will be able to reach the target mycobacteria thus representing an ideal delivery system to take phages close to their intracellular bacteria.	

### Nano-Emulsification

Emulsification is one such technique of encapsulation in which uniform emulsions of water in oil can be formed by adding mixture of microbes, cells, enzymes, and drugs along with a compatible polymer and further dispersing this mixture to a another phase of vegetable oil (such as canola, corn oil etc.) (Tadros et al., 2004; Mcclements and Rao, 2011). The emulsions may further be stabilized by addition of emulsifiers and stabilizing agents. Nanoemulsions thus offer a new method of encapsulating sensitive molecules such as proteins, enzymes, phages in a nanoporous matrix. The confinement of bio-molecules within nanoemulsions causes a change in the surrounding a_w_ (water activity) which imparts higher stability during storage period (Franklyne et al., 2019). Such multiple emulsion formulations can act as a suitable housing system shielding the phage from inactivation from immune system, outer proteolytic enzymes while maintaining their structural and functional activity (Balcão et al., 2014; Esteban et al., 2014; Sawant et al., 2017).

Nanoemulsions offer unprecedented uniformity which enables controlled drop morphology and uniform shape (Abate et al., 2010; Wang et al., 2011; Vladisavljević et al., 2017). The best part is that variation among the drop sizes is negligible (<3%), yield of 10,000 drops formed per second is remarkable and high efficiency is achievable for phage encapsulation (Matochko et al., 2012; Vinner and Malik, 2018). The process is performed in a geometrically restricted environment consisting of micro and nano-level quantities of fluids and bringing two or more immiscible fluid streams into contact with one another either following in a co-flow pattern (streams are parallel) or cross flow pattern (streams meet at an angle) or flow-focussing pattern. Due to constriction, streams get accelerated and this causes it to break into drops (Zhao, 2013; Ran et al., 2017).

By microfluidic method, conventional multiple emulsions such as W_1_/O/W_2_ emulsion (with a middle phase which is immiscible with inner and outer phase) can be easily fabricated with high precision and high control over drop size and shape. A typical W1/O/W2 emulsion therefore consists of drops of inner water phase dispersed within oil phase which are further dispersed in outer water phase W2 (Kanouni et al., 2003; Dickinson, 2011). Next, is a version of this, in which one can control the number of inner drops encapsulated within the large drop (Chu et al., 2007; Deng et al., 2014; Azarmanesh et al., 2019). This variation can be highly useful for encapsulation of more than one type of phage i.e., in phage cocktails possible within a single large drop. Each phage type is present in different inner drop microenvironment thus allowing precise control over number and content of individual inner drops.

In addition, microfluidic approach also allows synthesis of multiple emulsion drops with concentric multiple shells around the core drop. Depending on the number of immiscible phases, it is possible to prepare different number of shells within the main drop. For example, we can have triple shells such as W1/O2/W3/O4 or even quadruple shells (Abate et al., 2010; Malik et al., 2017; Vladisavljevic et al., 2017). Droplets with multiple onion like inner shells enable co-encapsulation of multiple active components (e.g., phage and antibiotic co-encapsulation or phage and antibacterial peptide or lysine co-encapsulation) and sequential release as per the desired choice. Such multiple shelled droplets enable to give maximum protection in case of highly sensitive phage or phage enzymes from outer stress environment. For example, the outer shell may protect phage from gastric pH as being acid resistant. Then, the next inner shell may be suitable for burst release for immediate action and release of high numbers of phages to immediately contain the bacterial population. The last or inner shells may be suited for sustained phage release for maintain a slow yet sustained release pattern especially essential for treating chronic infections and this may also avoid the need of repeated phage administration.

Next, we discuss few studies focussed on use of nanoemulsions based strategies for delivery of phages. Balcao et al. (2014) reported the nano-emulsification of a broad spectrum lytic phage i.e., phi-2/2, active against enteric *Salmonella* and *Escherichia coli* producing multiple emulsions called water-in-oil-in-water (W/O/W) emulsions. For this, the team first dissolved the phage in inner aqueous phase, then dispersed in lipid oily phase (melted lipid mix consisting of glycerol, Softisan, and soybean phosphatidylcholine and finally an external aqueous phase (Lutrol F68) was then added to produce stable lipid nanoballoons called Win/O/Wext dispersions of phage particles. Results showed that such nanoemulsions allowed long term storage of phi-2/2 (tested over 92 days) with no loss of phage infectivity while encompassing full stabilization of phage three-dimensional structure suitable for use in aerosolised forms. Such multiple emulsions with compartmentalized internal structure represents a better strategy allowing to carry both polar and nonpolar molecules with higher control over release of the therapeutic molecule. On similar grounds, Rios et al. (2017) also developed aqueous core lipid nanodropletes using W/O/W multiple emulsion platform for encasing broad spectrum lytic *P. aeruginosa* specific phage. These stable nanoemulsions (called ME10 and ME1000) were further used to develop isotonic derivatives for administering the phage via nebulisation route to treat pulmonary pneumonia allowing the targeted release of phages directly at required site. Results showed 90% phage encapsulation efficiency of ME1000. The isotonic derivatives so formed when tested showed no cytotoxic/genotoxic effect on A549 and V79 cell lines thus proving safe for *in vivo* use. Studies have also demonstrated enhanced antibacterial activity seen following encapsulation of phage K in oil-in-water nano-emulsions when compared to non-encapsulated preparations (Esteban et al., 2014). In this study, nano-emulsions were produced by a unique thermal phase inversion process using 5% (w/w) soybean oil as the organic phase and BrijO10 and SM buffer as aqueous phase incorporating phage K. These stable nanoemulsions showed enhanced antibacterial activity as shown by complete lysis of three *S. aureus* strains (strains H560, H325, and Btn766) within the first 4 hours with concentration dropping to zero. Bacterial regrowth for *S. aureus* H560 started at 8 h and for H325 and Btn766 at about 12 h with phage in plain SM buffer while this did not occur with bacteriophage nanoemulsion formulations. All these strains showed significantly reduced growth in terms of O. D as well. When strains were grown in presence of 1:1 diluted emulsion there was a decrease of 13% O. D for strain H560, 21% reduction in strain H325, 55% reduction seen in strain Btn766 as compared to O. D in tryptone soy broth (TSB). Also, these nanoemulsions were more stable showing no significant drop in lytic efficiency when stored for 10 days either at room temperature or at 4°C.Moreover, such oil-in-water nanoemulsions exhibit enhanced transdermal penetration into deeper layers with higher biocompatibility with the skin tissue due to lesser amount of surfactant required for their preparation (Bouchemal et al., 2004; Azeem et al., 2009). This advocates use of nano-emulsified phage preparations for use in superficial and deep seated skin infections and wound applications/dressings allowing sustained release of active phages.

### Nanofibers: A New Delivery Platform

Nanofibers represent another important outcome of nanotechnology in which nano-sized continuous fibers are produced with controlled surface morphology by a process called “Electro-spinning”. Briefly, the process of electrospinning involves a polymer solution filled in a syringe which is then forced through the syringe needle in form of a drop. To this drop, very high voltage is applied that leads to deformation from round to conical forms (i.e., Taylor cone effect) and finally as the voltage exceeds a threshold value, there occurs formation of one or more jets that travel to a metal collector where the solvent evaporates and formation of nano-sized fibers initiates at the surface of the collector (Teo and Ramakrishna, 2006; Bhattarai et al., 2018; Shahriar et al., 2019). These nanofibers enjoy the properties of a high surface area to volume ratio, enhanced flexibility, ease of surface functionalities and good mechanical performace and resistance (Gugulothu et al., 2019; Dos Santos et al., 2020). The best thing is that a large number of bioactive molecules and even live cells (e.g., antibacterial, anti-inflammatory, anti-cancer drugs, nucleic acids, DNA, enzymes, vitamins, proteins, probiotics, viruses, and algae etc.) can be mixed with the polymer solution during the electrospinning process leading to formation of electrospun nanofibers while incorporating the drug molecule with high efficiency and thus acting as a suitable delivery system (Potrc et al., 2015; Torres-Martínez et al., 2018; Monfared et al., 2020). Also, one can optimise the process of nanofiber formation by controlling the parameters during synthesis time so as to change fiber size, thickness, shape, porosity, conductivity, degradation rates, times, and response etc. as per the ideal requirements (Xue et al., 2019). A range of polymers [poly (lactic-co-glycolic acid) (PLGA), chitosan, polyvinyl alcohol, poly (ε-caprolactone), Poly ethylene oxide (PEO), cellulose, alginate, collagen, and elastin etc.) have been used for producing nanofibers giving a wide option for selection and moreover, biodegradable polymers used are highly bio-compatible and less immunogenic (Bonino et al., 2011; Mengistu Lemma et al., 2016; Yew et al., 2018; Gugulothu et al., 2019).

**FIGURE 2:**
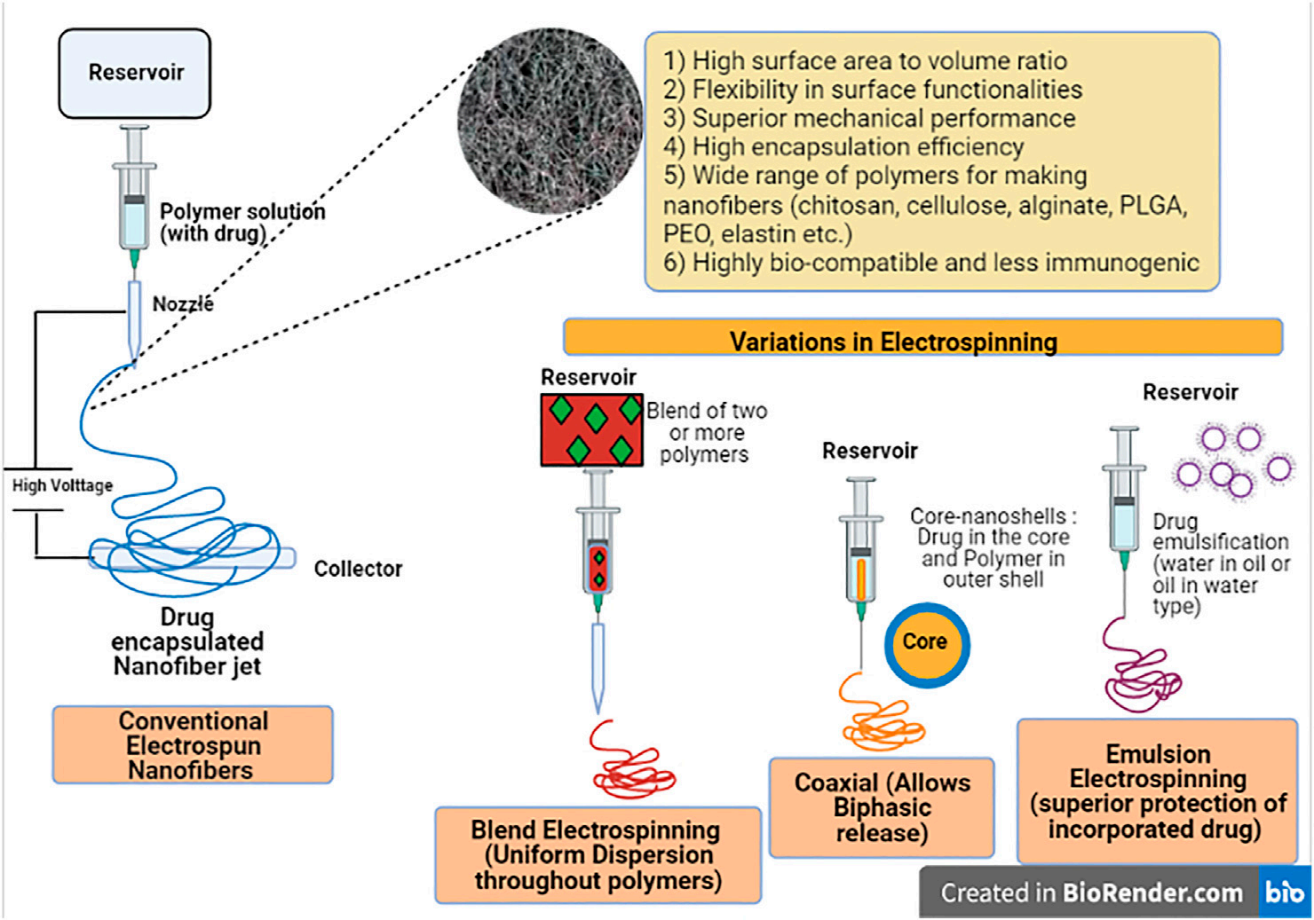
Diagrammatic illustration of Electrospun Nanofibers and its modifications as the new drug delivery platform.

Eletrospinning process can be manipulated in several ways to produce varying types of nanofibers (Alghoraibi and Alomari, 2018; Xue et al., 2019). Firstly, we have simple or basic electrospinning done with a single polymer allowing drug to be embedded evenly within the nanofibers. If drug loaded nanocarriers are mixed with the polymer, one will obtain nanocarriers attached to the outside of the fibers. Next, is the blend of two or more polymers,

which we refer as blend electrospinning that ideally helps in improved dispersion. Then, we have coaxial electrospinning process in which core-shell fibers are formed with drug within the core and polymer woven in the outer shell. Such coaxially electrospun nanofibers allow biphasic release pattern with an initial burst release from the core-sheath followed by extended periods of sustained release while many sensitive biomolecules are far better shielded within the core-shells than basic electrospinning (Lu et al., 2016; Xue et al., 2019). These core-shell nanofibers can either be multi-matrix or can be reservoir type depending on whether the drug is loaded in both layers or just within the core layer only. Besides this, there is emulsion electrospinning in which the drug forms emulsions either water-in-oil or oil-in-water types with formation of core–shell structured nanofibers. The degradable polymer is first solubilized in a proper organic solvent forming a continuous phase and the active drug is dissolved in the aqueous solution leading to formation of the water phase (Haider et al., 2018; Kumar et al., 2019). Such nanofibers offer superior protection of the incorporated drug from inactivation due to external stress conditions. Finally, there is gas jet type of modification, in which the process combines electrospinning with a gas jet device that allows for formation of highly uniform and ultra-fine nanofibers (Kenry and Lim, 2017). With all these newer modifications (as depicted in Figure 2), electrospun nanofibers can be produced to fit into any of the classes such as immediate drug release nanofibers, biphasic drug release (initial high burst and later sustained release) nanofibers, prolonged drug release nanofibers and stimulus-activated drug-release nanofibers (i.e., release in response to external stimuli when they meet a particular pH or temperature etc.)

### Nanofibers and Therapeutic Phage Delivery

Nanofibers so produced can be conjugated with range of active functional agents and lytic phages represent one of them. The biodegradable polymers are the best choice matrices for the deposition of phage within these fibers. Studies on the use of electrospun nanofibers for delivery of phages are presently scarce but the results are encouraging. Salalha et al. (2006) showed the successful basic electrospun nanofibers for encapsulation of T4, T7, and lambda phage in poly-vinyl alcohol (PVA) polymer resulting in maintenance of good viability for at least 3 months at 4°C. Although *in vivo* efficacy of these nanofibers was not studied, but this study offered the first hope of electrospinning as an ideal solution against phage viability losses. Similarly, nanofibers embedded with phage provided protection which was further improved with addition of storage media (SM) buffer and sucrose over an extended period of 8 weeks at 20°C (Koo et al., 2016). However, one drawback associated was loss of lytic activity of phages once released from such nanofibers and the possible reason for this is the rapid dehydration of bacteriophage during the spinning process (Lee and Belcher, 2004). Although addition of trehalose, sucrose, and mannitol exhibit a protective effect from high voltage and spinning process but loss of activity was still observed (Dai et al., 2014).

Korehei and Kadla (2013) addressed this issue by using two different electrospinning processes: emulsion and coaxial electrospinning. In emulsion electrospinning, pre-encapsulation of bacteriophage T4 in alginate matrix was first done followed by electrospinning whereas in coaxial electro spinning process, the phage was allocated to the core of the fibers. Emulsion electrospun fibers with T4 bacteriophages incorporated in alginate capsules, provided a shielding barrier against rapid dehydration stress with only a slight drop in phage activity observed. Further, the most promising results were obtained when coaxial nanofibers with T4 bacteriophages were formed as full bioactivity was maintained. Coaxial spinning produces continuous core/shell morphology with a more uniform distribution of phage in the core of the fibre and no drastic change in the osmotic environment leading to retention of full lytic activity.

Another important application of nanofibers is their use in wound dressing allowing sustained release of phages at wound site while maintain complete infectivity. This was reported by Nogueira et al. (2017) in which the team developed a novel method of covalent immobilization via amide linkages of vB_Pae_Kakheti25 bacteriophage capsid on poly-caprolactone (PCL) nanofibers with oriented phage tails ready to interact with the bacteria.

These immobilised phage nanofibers showed as high as six log reduction (99.9%) in *P. aeruginosa* counts while utilising PCL polymer’s properties of skin regeneration, high elasticity and resistance to breakdown from skin enzyme etc. Such systems represent as an ideal approach for sustained release of phage from the wound dressing while promoting wound healing and skin regeneration, a favourable two in one approach for treating chronic skin ailments (Sarhan and Azzazy, 2017).

One important application is the use of stimulus activated smart release nanofibers for release of phages as and when required. This is especially suitable in oral delivery of phages. These smart release nanofibers would not allow the release of phages at low pH of stomach thus shielding them during gastric transit. Once the phage loaded nanofibers reach the intended site (e.g., colon) and meet the desired pH, phage release will be initiated in desired format (burst or sustained). Delivery of antibiotic and anticancer drugs, probiotics using cellulose smart has been reported (Demirci et al., 2014; Hujaya et al., 2018; Singh et al., 2018). Such results advocate more studies on use of pH responsive phage embedded nanofibers to address the issue of viability and titer loss during gut transit.

### Stimuli-Responsive Nanocarriers

Another technique for drug delivery is the smart release platforms which allow the release of active drugs at the desired site in response to an external stimuli. This stimuli can be a particular pH, temperature, or a molecule released by bacteria, an enzyme, protein, cytokine, and signalling molecule etc. The stimuli triggered release concept has been the subject of interest as it allows control over the dosing releasing the drug where, and when it is desired achieving high local concentrations (Webber, 2016; Wells et al., 2019; Abdo et al., 2020). Let’s discuss the commonly used smart release DDS available and its outcome on phage delivery.

One such synthetic polymer widely used is Eudragit^®^. This belongs to family of pH responsive polymethacrylate polymers that respond to changes in pH and come in different grades (Nikam et al., 2011; Thakral et al., 2013). For example, Eudragit^®^ L100 dissolves at pH six while Eudragit^®^ S100 at pH 7. Hence, use of such polymers as encapsulating matrix will enable the drug to be released only at the right time and at the right site when the desired pH is met. Eudragit L and S are two forms that form films/coatings resistant to low gastric pH thereby allowing the drug to easily bypass the stomach and reach the intestine. These films are soluble in intestinal fluid at pH six and seven whereby they release the active drug (Vibhooti et al., 2013; Mehta et al., 2013). This same scheme can be employed for encapsulating

phages and protecting them from gastric pH allowing release only at near neutral pH at the desired intestinal target site essential for success of oral phage therapy targeting various enteric infections. Similarly, Eudragit RS100 and E100 have been used for triggered release at acidic pH such as during vaginal delivery (Leyva-Gomez et al., 2018). There are recent studies that have explored the potential of this pH responsive polymer for triggered phage release at desired pH. Although these studies have reported the formation of Eudragit microspheres and microparticles, the encouraging results of these studies on enhancing phage stability advocate scaling down the process for formation of nanospheres and nano-coatings. Few studies are worth mentioning. Vinner at al. (2017) reported the encapsulation of *C. difficile* specific phage CDKM9 in EudragitS100 with and without alginate. By adopting a unique microfluidic system using flow focussing glass microcapillary device, the phage preparations were encased within the uniform core-shell microspheres so formed. Results showed that the phage titers did not drop when such phage loaded microparticles were exposed to simulated gastric fluid environment of pH two for 3 h and phage release was triggered with high titers of viable phage moving out at the desired pH 7. Same group further developed a unique scalable low shear membrane emulsification process through which they produced uniform pH-responsive microparticles containing *E. coli* phages encapsulated in EudragitS100 and alginate (Vinner et al., 2019). The encapsulated phages were completely stable and well-protected from gastric pH stress, easy inactivation from bile fluids, enzymatic action etc. Such microcapsules are very apt and suitable for delivering high numbers of viable phage at the target site in the intestinal system. Moreover, development of scalable processes such as the membrane emulsification process is highly required as it represents a potential platform for producing large scale quantities (following GMP norms) of such phage loaded microspheres for oral solid dosage forms and widespread clinical testing.

Another polymer that shows triggered response to temperature as the stimuli, is Poly (N-isopropylacrylamide) (PNIPAM). It is a thermally responsive polymer that undergoes a reversible phase transition in response to changes in external temperature. There occurs a sharp phase transition when the polymer is exposed to water at 32°C called lower critical solution temperature (LCST) phase transition whereby gel changes from swollen hydrated state to a shrunken or collapsed state, losing about 90% of its volume (Heskins and Guillet, 1968; Lima et al., 2016; Pal et al., 2019). There has been limited study on use of this polymer for developing drug delivery platform for phages and hence this needs further exploitation.

Hathaway et al. (2015) utilised PNIPAM nanosphere co-polymerized with allylamine (ALA) for development of smart delivery system of phages at wound site allowing phage loaded nanospheres to collapse (allowing phage release in high numbers) at an elevated temperature during an active bacterial infection site. The formulated PNIPAM-co-ALA nanoparticles were anchored to non-woven polypropylene to simulate a wound dressing and then soaked with phage K solution (10^9^ PFU/ml) for 4 h at 25°C. This phage loaded thermal responsive PNIPAM-co-ALA nanospheres collapsed at 37°C releasing the phage K leading to lysis of clinical *S. aureus* isolate ST228. However, at 25°C, the system remained intact with no release of phages with no decline seen in the bacterial counts. In another study by the same group, Hathaway et al. (2016) reported the successful encapsulation of two agents i.e., phage endolysin CHAPK and the bacteriocin lysostaphin in PNIPAM nanoparticles and then tested their potency against control of MRSA mediated wound infections. PNIPAM nanoparticles showed effective release of the enzybiotic cocktail in a temperature controlled manner. CHAPK/lysostaphin released from PNIPAM showed significant bacterial lysis with four log reduction at 37°C while growth was maintained at temperature of 32°C in case of uninfected skin temperature. In addition to targeting wound infections, such nano-coatings may prove highly useful in case of implants and catheters whereby any initial infection of foreign implants will cause a thermal change and this will trigger phage release at the implant site. Moreover, phage and antibiotic can be encapsulated together in the nanospheres and further coated onto implants for exploring the dual potential of the two agents as reported by Kaur et al. (2016) against orthopaedic implant infections.

## Conclusion

Modern phage therapy has come a long way and there has been some major case studies reporting successful use of phage therapy (on compassionate basis) in treating chronic and refractory infections. Although direct indication of success of these human studies using nano-based advances has not been tested or reported, but looking at the success stories in individual patients, the advocated use of improved nano-based delivery systems in future will definitely further enhance the treatment success rates. There has been recent reports of case studies whereby phage cocktail therapy has shown improvement of chronic respiratory infections from drug resistant strains (*P. aeruginosa, Acinetobacter baumannii, Burkholderia complex, Achromobacter xylosoxidans etc.*) seen in CF patients (Duplessis et al., 2019; Aslam et al., 2020; Lebeaux et al., 2021). In such cases, the use of inhalable liposomal phage formulations for nebulised administration may be a step ahead for further maintaining phage titers and protection from local immune attack thus enhancing the treatment outcome in such difficult to treat pulmonary infections (Bassetti et al., 2020). Also, use of multiple nano-emulsions and core shell nanofibers may offer a better compartmentalization and sustained release of phage cocktails. Similarly, successful case reports of treatment of diabetic toe wound infections by local application of *S. aureus* phage cocktail (Fish et al., 2016) and foreign body infections such as prosthetic implant infection whereby phage cocktails were locally injected into the joint cavity have been reported (Ferry et al., 2018; Nir-Paz et al., 2019). Here, the stimuli-responsive nano-carriers and electrospun nanofibers may be an ideal strategy to enhance the bioavailability of delivered phages in the affected wound site and implant area in a sustained manner for longer duration. Also, such smart release systems will enable the timely release of the drug as soon as bacterial multiplication initiates (causing change in stimuli such as temperature or pH) thus able to arrest the infection or biofilm formation process at the initial stages itself.

In the present scenario, where bacterial resistance has become an alarming crisis responsible for the increased number of deaths due to infections not responding to antibiotic treatment, phage therapy definitely offers a ray of hope. It needs to be strongly promoted and worked on with focussed approach towards addressing the major limitations. The present review has detailed out the major pharmacological barriers and the new developments and solutions offered by nanotechnology. These advances in nano-delivery based strategies exhibit strong potential in enhancing/improving the therapeutic and clinical outcome of the delivered phage while overcoming many of the major drawbacks that are still unmet. This advocates further research in this direction so that phage therapy can see wider clinical success and faster progress.
